# Quantitative Assessment of the Polymorphisms in the *HOTAIR* lncRNA and Cancer Risk: A Meta-Analysis of 8 Case-Control Studies

**DOI:** 10.1371/journal.pone.0152296

**Published:** 2016-03-24

**Authors:** Tian Tian, Chunjian Li, Jing Xiao, Yi Shen, Yihua Lu, Liying Jiang, Xun Zhuang, Minjie Chu

**Affiliations:** 1 Department of Epidemiology and Biostatistics, School of Public Health, Nantong University, Nantong, Jiangsu, China; 2 Analysis and Testing Center of Nantong University, Nantong, Jiangsu, China; Duke Cancer Institute, UNITED STATES

## Abstract

HOX transcript antisense intergenic RNA (*HOTAIR*) is a long non-coding RNA (lncRNA) that functions as an oncogenic molecule in different cancer cells. Genetic variants of *HOTAIR* may affect the activity of certain regulatory factors and further regulate the aberrant expression of *HOTAIR*, which might be underlying mechanisms that affect tumour susceptibility and prognosis. Recently, several studies have been performed to examine the possible link between polymorphisms in *HOTAIR* and cancer risk; however, the results have been inconclusive. Therefore, we performed a meta-analysis to estimate the associations between *HOTAIR* polymorphisms (rs920778, rs4759314 and rs1899663) and cancer risk. Eight studies comprising 7,151 cases and 8,740 controls were included in our study. Overall, no significant associations between the *HOTAIR* polymorphisms (rs920778, rs4759314 and rs1899663) and cancer risk were observed. However, in further stratified analyses, the variant T allele of rs920778 exhibited a significant increased risk of developing digestive cancers (dominant model: OR = 1.44; 95% CI = 1.31–1.59). These findings provided evidence that *HOTAIR* rs920778 may modify the susceptibility to certain cancer types. Further studies incorporating subjects with different ethnic backgrounds combined with re-sequencing of the marked region and functional evaluations are warranted.

## Introduction

Cancers are the leading cause of deaths worldwide. According to WHO estimates, there were 14.1 million new cancer cases in 2012, and over 20 million new cancer cases will be expected annually as early as 2025, indicating an ever-increasing global cancer burden [1]. Generally, cancers are considered to be multifactorial diseases, and the occurrence of cancers are related to environmental, genetic and lifestyle factors. Among these factors, the genetic factors are of particular interest, especially because recent genome-wide association studies (GWAS) and next-generation sequencing (NGS) have greatly broadened our understanding of the genetic variations that confer risks for cancers.

Long non-coding RNAs (lncRNAs) are a type of non-coding RNA (ncRNA) that contain more than 200 nucleotides and do not encode proteins but have pivotal roles in numerous biological functions. Recent reports suggest that aberrant expression of lncRNAs might play important roles in the development and progression of tumours [2,3,4] that are likely mediated through changes at the chromatin, transcriptional or post-transcriptional levels that influence target gene expression [5]. Additionally, GWASs have successfully identified several lncRNA polymorphisms that are associated with the risks of developing different types of cancer. However, similar to the other reported GWAS, the majority of the lncRNA SNPs that have been identified by GWAS have been mapped to intergenic regions or introns that do not encode proteins, and the potential functions of these SNPs in the pathogenesis of cancer remain undefined. Furthermore, due to the stringent screening criteria of GWASs, some putative causal lncRNAs and variants that are associated with carcinogenesis may have been be largely ignored. Thus, additional efforts directed toward candidate lncRNAs that function in the development of cancer may be expedient to uncover part of the missing heritability. One such lncRNA is the HOX transcript antisense intergenic RNA (*HOTAIR*), the function of which has been demonstrated to be closely related to the development and progression of some cancers [6,7,8].

To date, the relationship between the aberrant expression of *HOTAIR* and cancer prognosis has been explored by many researchers. Moreover, meta-analyses have demonstrated that the aberrant expression of *HOTAIR* may serve as an indicator that predicts poor prognoses both in cancer overall [9] and in particular types of cancers (e.g., digestive system cancers and oestrogen-dependent malignant tumours) [10,11,12]. Additionally, a meta-analysis performed by Cai et al. revealed that the overexpression of *HOTAIR* is significantly associated with lymph node metastasis in cancer patients, which might further affect cancer prognoses [13].

In 2015, Bayram S et al. demonstrated that rs920778 SNP of *HOTAIR* is significantly associated with advanced TNM stage, distant metastasis and poor histological grade in breast cancer patients, which indicates that this polymorphism may be associated with breast cancer prognosis [14]. Similarly, it has been reported that another SNP in *HOTAIR* (rs12826786) is associated with the clinicopathological features involved in gastric cardia adenocarcinoma progression [15]. Additionally, relationships of *HOTAIR* polymorphisms (including rs4759314 and rs920778) with the expression of *HOTAIR* and various cancer risks have been observed [15,16,17,18]. Moreover, Zhang et al. reported that SNP rs920778 in *HOTAIR* may alter the activity of a novel intronic *HOTAIR* enhancer [17]. Thus, it is biologically conceivable that the genetic variants of *HOTAIR* may affect the activities of certain regulatory factors and further regulate the aberrant expression of *HOTAIR*, which might be one of the underlying mechanisms that affect tumour susceptibility and prognosis. As expected, the relationships *HOTAIR* of polymorphisms with sensitivities to cancers have attracted much interest [14,15,16,17,18,19,20,21]. However, the results of the studies that have explored this association are inconclusive. For example, a previous study report that the rs920778 variant genotype significantly increases the risk of gastric cancer in Chinese people [16]; however, among Turkish people, the same variant exhibited no significant association, and the effect values were even in the opposite direction relative to the previous studies of Chinese populations [19]. Therefore, based on all of the currently published data, we performed a meta-analysis to more precisely characterize the associations of *HOTAIR* polymorphisms (rs920778, rs4759314 and rs1899663) with cancer risk.

## Materials and Methods

### Identification and eligibility of the relevant studies

**Fig 1:**
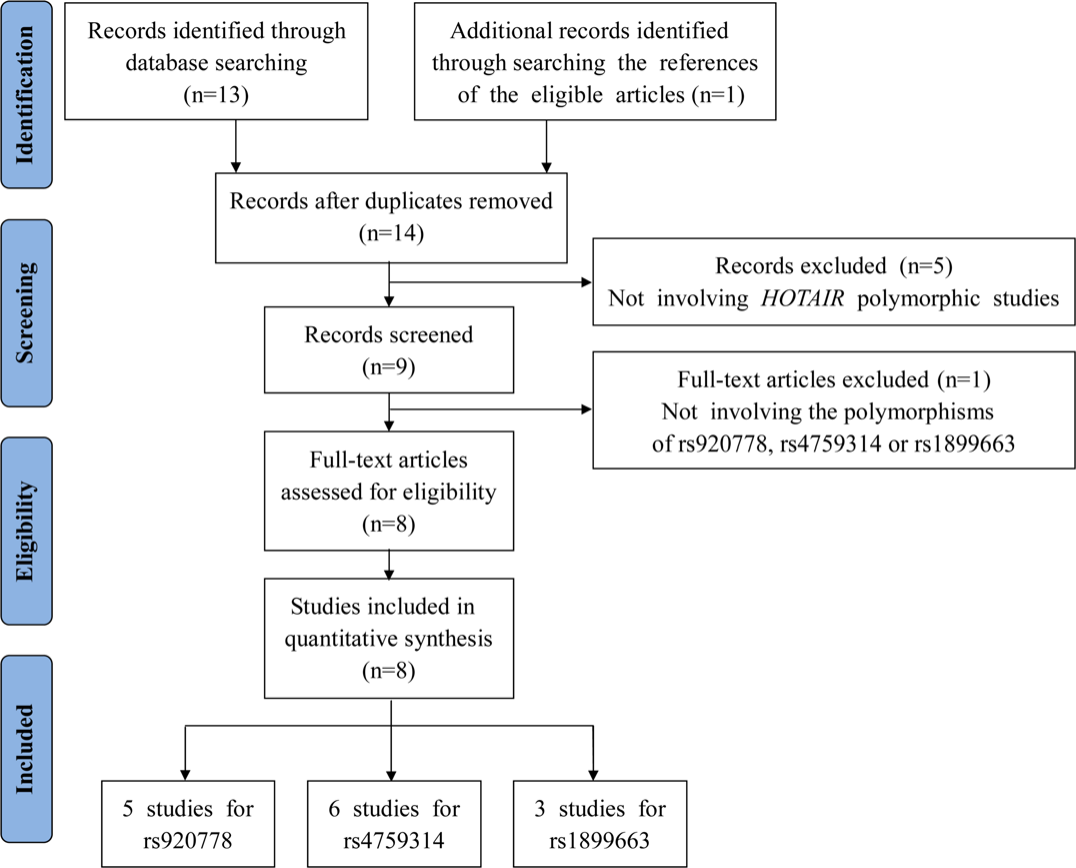
Flow diagram of the study selection process.

A comprehensive literature search of PubMed and Embase up to November 30, 2015 was performed using the following keywords: ("*HOTAIR*" or "HOX transcript antisense intergenic RNA") and ("cancer", "carcinoma", "tumor", "tumour", or "neoplasm") and ("polymorphism", "variation", "variant", or "mutation"). The references in retrieved articles were also reviewed for possible inclusion. Only publications written in English with available full-text articles were included in this meta-analysis. Studies were included if they met the following eligibility criteria: (1) case-control studies focused on the relationship between *HOTAIR* polymorphisms and any type of cancer, (2) more than two articles for each studied *HOTAIR* polymorphism, (3) available information about the genotype frequency of each included *HOTAIR* SNP (i.e., rs920778, rs4759314 or rs1899663), and (4) published as a full paper in English. The main reasons for the exclusion of studies were the following: (1) not focused on cancer risk, (2) did not study the *HOTAIR* SNPs (rs920778, rs4759314 or rs1899663), (3) did not report the relevant genotype frequency data, (4) not published in English, and (5) non-human research. Ultimately, a total of 8 articles including 7,151 cases and 8,740 controls were included in this meta-analysis (**Fig 1**).

### Data extraction

Two investigators (T.T. and L.J.) independently extracted the data and reached consensus regarding all of the items. The following information was sought from each article: the first author’s name, year of publication, country of origin, ethnicity, type of cancer, numbers of cases and controls, genotyping platform and genotyped SNPs. We categorized the different ethnicities as Caucasians and Asians.

### Statistical analysis

The risk of cancer associated with each *HOTAIR* polymorphism was estimated for each study using the odds ratio (OR) and its 95% confidence interval (95%CI). The between-study heterogeneity was examined with a chi-square-based *Q* statistic test, and *P* ≤ 0.05 was considered as statistically significant. We pooled the results using fixed-effect models when the heterogeneity between studies was absent. Otherwise, a random-effects model was selected. Subsequently, we evaluated the risks of the heterozygous and variant homozygous genotypes relative to the wild-type homozygous genotype and then evaluated the risks of the combined heterozygous and variant homozygous genotypes relative to the wild-type homozygous genotype while assuming the dominant effects of the variant allele. For rs920778, we also performed a stratification analyses based on ethnicity (divided into Caucasians and Asians) and cancer types. Funnel plots and Begg’s test were utilized to evaluate the publication bias. All analyses were performed using the Stata version 12.0 software (Stata Corporation, College Station, TX, USA).

### Functional annotation based on publically available databases

The SNPs in high LD (*r*^2^ ≥ 0.80) with the marker SNP rs920778 were identified according with SNAP 2.2 (http://www.broadinstitute.org/mpg/snap/ldsearch.php/). Expression quantitative trait loci (eQTL) analysis was performed based on the Genotype-Tissue Expression project (GTEx) database (http://www.gtexportal.org/home/). The influences of the SNPs on miRNA binding were predicted with an online service (http://www.bioguo.org/miRNASNP2/). Additionally, other functional annotation results were derived from the ENCODE database (http://genome.ucsc.edu/ENCODE/).

## Results

### Characteristics of the published studies

After the application of the strict screening criteria, 8 articles that included a total of 7,151 cases and 8,740 controls harbouring gastric cancer, breast cancer, colorectal cancer and oesophageal squamous cell carcinomas (ESCCs) were ultimately included in the current quantitative analysis. The general characteristics of the included studies are listed in **Table 1**, and the excluded studies are listed in **S1 Table**. Among the included studies, six studies were conducted in Asians (Chinese) populations, and two studies were conducted in Caucasian (Turkish) populations. The publication years of the included studies were between 2014 and 2015, and the range of the sample sizes was 245 to 4,248. A total of four articles reported the effects of *HOTAIR* polymorphisms in gastric cancer, two in breast cancer, one in oesophageal squamous cell carcinoma (ESCC), and one in colorectal cancer. Among the studies that explored the relationships of *HOTAIR* SNPs with cancer risk, five focused on the rs920778 SNP, six on the rs4759314 SNP and three on the rs1899663 SNP. Genotyping was performed using TaqMan in 4 studies, PCR-RFLP in 3 studies and CRS–RFLP in 1 study. The distributions of the genotypes and alleles of the *HOTAIR* polymorphisms (rs920778, rs4759314 and rs1899663) in the individual studies are listed in **S2**–**S4 Tables**.

**Table 1:** Characteristics of the studies included in the meta-analysis.

First Author	Year	Country	Ethnicity	Type of cancer	Case/Control	Platform	Genotyped SNPs
Zhang	2014	China	Asian	ESCC ^a^	2098/2150	PCR-RFLP	rs920778, rs4759314, rs1899663
Bayram	2015	Turkey	Caucasian	gastric cancer	104/209	TaqMan	rs920778
Pan	2015	China	Asian	gastric cancer	800/1600	PCR-RFLP	rs920778, rs4759314, rs1899663
Xue	2015	China	Asian	colorectal cancer	1734/1855	TaqMan	rs4759314
Du	2015	China	Asian	gastric cancer	1275/1646	TaqMan	rs4759314
Guo	2015	China	Asian	GCA ^b^	515/654	PCR-RFLP	rs4759314
Bayram	2015	Turkey	Caucasian	breast cancer	123/122	TaqMan	rs920778
Yan	2015	China	Asian	breast cancer	502/504	CRS–RFLP/PCR-RFLP	rs920778, rs4759314, rs1899663

^a^ oesophageal squamous cell carcinoma (ESCC)

^b^ gastric cardia adenocarcinoma (GCA)

### Quality assessments of the included studies

The methodological quality of each included study was evaluated using the Newcastle-Ottawa quality assessment scale (NOS). Using this method, each study was judged on standard criteria and subsequently categorized based on three factors: selection, comparability, and exposure. Summary scores ranging from 0 to 9 points were calculated, and higher scores indicate lower risks of bias (**S5 Table**).

### Quantitative synthesis

**Fig 2:**
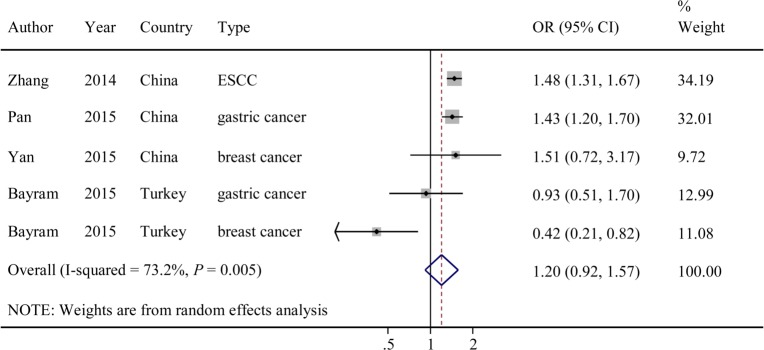
Forest plot of OR with 95%CI for the *HOTAIR* rs920778 with cancer risk under dominant model.

The evaluations of the associations of rs920778 with cancer risks are presented in **Table 2**. Overall, the T allele variant exhibited no significant association with cancer risk in any of the tested models (CT versus CC: OR = 1.11; 95% CI = 0.86–1.44; *P* = 0.014 for the heterogeneity test, *I*^*2*^ = 67.8%; TT versus CC: OR = 1.55; 95% CI = 0.84–2.85; *P* = 0.000 for the heterogeneity test, *I*^*2*^ = 85.8%; dominant model: OR = 1.20; 95% CI = 0.92–1.57, *P* = 0.005 for the heterogeneity test, *I*^*2*^ = 73.2%; **Fig 2).**

**Table 2:** Summary ORs of the *HOTAIR* rs920778 polymorphism and cancer risk.

Variables	Studies	Sample size	CT versus CC			TT versus CC			Dominant model		
			OR(95%CI)	*P* ^a^	*I* ^*2*^	OR (95%CI)	*P* ^a^	*I* ^*2*^	OR(95%CI)	*P* ^a^	*I* ^*2*^
Total	5	8,212	1.11(0.86–1.44)	0.014	67.8%	1.55(0.84–2.85)	0.000	85.8%	1.20(0.92–1.57)	0.005	73.2%
*Ethnicity*											
Asians	3	7,654	1.32(1.19–1.47)	0.938	0.0%	2.76(2.22–3.43)	0.403	0.0%	1.46(1.32–1.61)	0.953	0.0%
Caucasians	2	558	0.63(0.39–1.00)	0.064	70.8%	0.68(0.41–1.12)	0.199	39.4%	0.65(0.42–1.00)	0.080	67.4%
*Cancer type*											
digestive cancer ^b^	3	6,961	1.31(1.19–1.46)	0.564	0.0%	2.17(1.26–3.75)	0.008	79.4%	1.44(1.31–1.59)	0.339	7.6%
breast cancer	2	1,251	0.67(0.22–2.04)	0.032	78.1%	0.90(0.25–3.20)	0.017	82.4%	0.79(0.22–2.78)	0.012	84.3%

^a^ *P* for heterogeneity (a random-effects model was used when the *P* value for heterogeneity test was < 0.05; otherwise, a fixed-effect model was used.)

^b^ including gastric cancer and ESCC

We next evaluated the effect of the rs920778 polymorphism on cancer risk among the subgroups **(Table 2)**. In a stratified analyses, a significantly increased cancer risk was observed among Asians (Chinese; dominant model: OR = 1.46; 95% CI = 1.32–1.61; *P* = 0.953 for the heterogeneity test, *I*^*2*^ = 0.0%). In contrast, a significantly decreased cancer risk was observed among Caucasians (Turkish; dominant model: OR = 0.65; 95%CI = 0.42–1.00; *P* = 0.080 for the heterogeneity test, *I*^*2*^ = 67.4%). Additionally, because the majority (60%, 3/5) of the studies included in our meta-analysis involved digestive cancers (gastric cancer and ESCC), we then performed a separate analysis for digestive cancers. Interestingly, the rs920778 variant exhibited a significant association with an increased risk of digestive cancers (dominant model: OR = 1.44; 95% CI = 1.31–1.59; *P* = 0.339 for the heterogeneity test, *I*^*2*^ = 7.6%; **Table 2)**.

**Fig 3:**
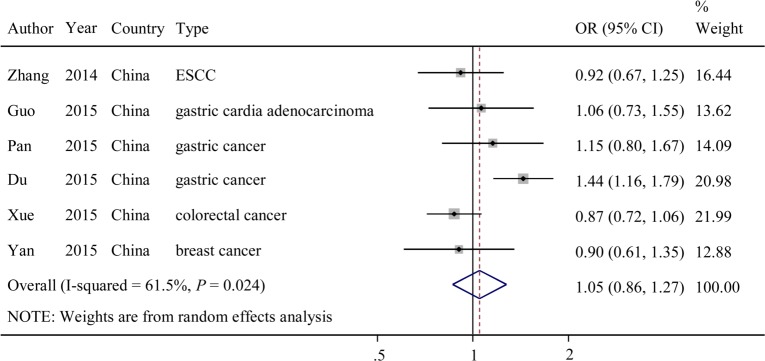
Forest plot of OR with 95%CI for the *HOTAIR* rs4759314 with cancer risk under dominant model.

**Fig 4:**
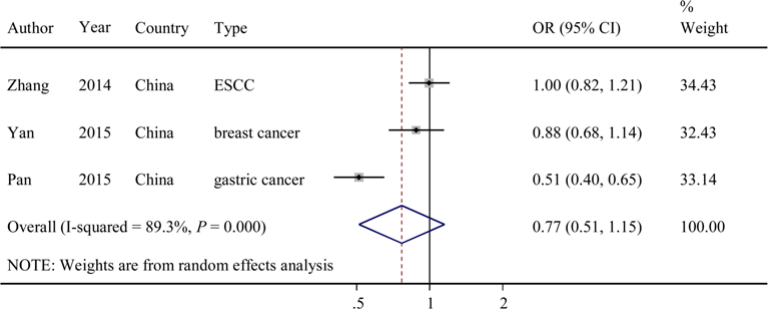
Forest plot of OR with 95%CI for the *HOTAIR* rs1899663 with cancer risk under dominant model.

The evaluations of the associations between the other 2 SNPs (rs4759314 and rs1899663) and cancer risk are presented in **Figs 3 and 4**. Overall, the G variant allele of rs4759314 exhibited no significant association with cancer risk (dominant model: OR = 1.05; 95% CI = 0.86–1.27, *P* = 0.024 for the heterogeneity test, *I*^*2*^ = 61.5%). The T variant allele of rs1899663 also exhibited no significant association with cancer risk (dominant model: OR = 0.77; 95% CI = 0.51–1.15, *P* = 0.000 for the heterogeneity test, *I*^*2*^ = 89.3%).

### Test of heterogeneity

For rs920778, significant heterogeneity was observed after the data were pooled (dominant model: *P* for heterogeneity = 0.005, *I*^*2*^ = 73.2%). (**Table 2**) When the subjects were stratified based on ethnicity, the heterogeneity obviously disappeared in the Asians (dominant model: *P* for heterogeneity = 0.953, *I*^*2*^ = 0.0%); however, heterogeneity was still present among the Caucasians (dominant model: *P* for heterogeneity = 0.080, *I*^*2*^ = 67.4%), which might have been due to the genetic heterogeneity between the different included ethnicities. Additionally, in stratified analyses based on cancer types, the heterogeneity was significantly reduced for the digestive cancers (dominant model: *P* for heterogeneity = 0.339, *I*^*2*^ = 7.6%).

### Sensitivity analysis

To test the stability of the rs920778 results, we performed sensitivity analyses by sequentially removing each eligible study (**S6 Table**). The study by Bayram et al. that focused on breast cancer was the major contributor of heterogeneity in the dominant model (*I*^*2*^ = 73.2%, *P* for heterogeneity = 0.005). After the removal of this study, the heterogeneity was significantly reduced (*I*^*2*^ = 0.0%, *P* for heterogeneity = 0.536). As expected, similar results were observed in the other genetic models (i.e., CT versus CC and TT versus CC) and indicated that the study by Bayram et al. that focused on breast cancer markedly changed the pooled OR.

### Publication bias

We used funnel plots and Begg’s test to evaluate potential publication biases of the studied literature. As illustrated in **S1**–**S3 Figs**, the shapes of the funnel plots were symmetrical, and a Begg’s test provided further statistical evidence for the absence of publication bias (*P* = 0.14 for rs920778, *P* = 0.85 for rs4759314, and *P* = 0.60 for rs1899663).

## Discussion

Recently, an increasing number of studies have investigated the associations of different *HOTAIR* expression levels with cancer survival, but fewer studies have focused specific attention on the relationships of *HOTAIR* polymorphisms with cancer susceptibility and survival. Nevertheless, the relationships of *HOTAIR* polymorphisms with cancer sensitivities have attracted much interest; however, the results are controversial. In the present study, we performed a meta-analysis by pooling 8 studies with totals of 7,151 cases and 8,740 controls and demonstrated that the T allele of rs920778 was associated with a significant increased risk of digestive cancers. However, the rs4759314 and rs1899663 variant alleles exhibited no significant associations with cancer risk.

The rs920778 SNP at 12q13.13 is located in intron 2 of *HOTAIR*. Based on public datasets and tools (**see Methods; S7 Table**), we performed further functional annotations of the marker SNP rs920778 and the SNPs that are in strong LD with rs920778 (*r*^2^> 0.8). Among the 11 SNPs that were found to be in strong LD with rs920778, significant genotype-specific effects of 5 SNPs (i.e., rs10783618, rs11170775, rs4759059, rs4237809 and rs2366150) on the mRNA expression of *HOTAIR* were observed (eQTL analysis). Subsequently, we explored the possible mechanisms by which these SNPs modulate the expression of *HOTAIR*. According to the ChIP-Seq data from the ENCODE database (http://genome.ucsc.edu/ENCODE/), a total of 10 SNPs were located in motifs that may affect the binding activities of numerous transcription factors, including EZH2, CTBP2, CHD1, ZNF143, SUZ12, TCF7L2, CTCF, RAD21, and YY1. The transcription factors mentioned above may be intimately connected with the occurrence and progression of many types of tumours in human. For example, enhancer of zeste homologue 2 (EZH2) is over-expressed in several human tumours and accounts for the aggressiveness and unfavourable prognoses of various tumours [22,23]. Furthermore, the suppressor of zeste-12 protein (SUZ12) is of great importance in the tumourigenesis of several human cancers [24,25] and is involved in the progression of non-small cell lung cancer via its role in promoting cell proliferation and metastasis [26]. Additionally, as predicted by a miRNA-binding analysis website (http://www.bioguo.org/miRNASNP2/), the rs7958904 SNP may affect the binding activity of hsa-miR-615. This miRNA is a newly identified tumour suppressor that can regulate the proliferation, migration, invasion, and apoptosis of various types of cancer cells [27,28]. Thus, these functional annotations suggest that the above-mentioned SNPs (including the marker SNP rs920778 and the tagged SNPs) might separately or jointly influence the aberrant activities of certain transcription factors or miRNAs and further affect the occurrence and progression of certain tumours through different mechanisms. However, experimental evidence validating this hypothesis is limited, and future functional studies are needed to clarify the possible mechanisms.

In this meta-analysis, we did not identify a significant relationship of the rs920778 SNP with cancer risk. However, according to the analyses stratified by population ethnicity, the rs920778 SNP was significantly associated with an increased risk of cancer in Asians (Chinese). In contrast, we found that the rs920778 polymorphism exhibited the opposite association with the risk of cancer in Caucasians (Turkish). The possible reasons for the different results between Asians and Caucasians are as follows. First, the difference may have resulted from differences in the genetic backgrounds of the studied populations. For example, based on the HapMap data (International HapMap Project), the allele frequencies of the rs920778 SNP are different between Asian and Caucasian populations. Second, the difference may owe to the utilization of different genotyping methods, which included PCR-RFLP, CRS-RFLP, TaqMan Real-Time PCR, etc. Third, compared with Asian populations, the sample sizes of the Caucasian populations might not have been sufficiently large to reach a convincing conclusion regarding the association of the rs920778 SNP with cancer risk. Additionally, the different types of cancers involved and random errors may also be potential reasons for the differences in the findings between Asians and Caucasians.

In 2014, Deng et al. conducted a meta-analysis that revealed that *HOTAIR* abundance might serve as a novel predictive factor for poor cancer prognoses [9]. The results of this study and our results both demonstrated significant relationships between *HOTAIR* and cancer. The findings of our study provided evidence that *HOTAIR* polymorphisms might modify cancer susceptibility. Unfortunately, in their meta-analysis of the prognostic value of *HOTAIR* in cancer, Deng et al. did not explore the associations of *HOTAIR* polymorphisms with cancer prognoses, and the relevant research on *HOTAIR* polymorphisms and cancer prognoses is limited. However, based on both findings and functional annotation results, we speculate that *HOTAIR* polymorphisms might regulate the expression of *HOTAIR* and further affect cancer development and progression via certain underlining mechanisms, which may open new avenues for the prevention and treatment of cancer.

The strength of this meta-analysis is that we systemically reviewed the relationships between *HOTAIR* polymorphisms and tumour susceptibility for the first time, and we identified different associations of the rs920778 SNP with cancer risk in different ethnic populations. Additionally, the well-designed functional annotations that further verified our findings are another strength of this study. However, there are also some limitations that need to be addressed. First, significant heterogeneity between studies was observed. Among the 8 published studies included in our meta-analysis, some of the studies were population-based, while others were hospital-based. Second, we did not search Chinese databases, which will result in publication bias. Third, in some of the studies, detailed information (e.g., age, gender, smoking status, and alcohol consumption) was not provided, which limited further stratification analyses. Additionally, if we had been able to acquire more detailed information, we would have achieved more precise estimations by adjusting for other potential covariates. Finally, for this study, we collected only 8 reports. The majority of the subjects were Asians, and the number of Caucasian subjects included in this study was relatively small. Thus, well-conducted large-sample studies are needed to further explore the cancer risks associated with *HOTAIR* SNPs, especially in Caucasians.

## Conclusions

This meta-analysis provided evidence that *HOTAIR* rs920778 may modify the susceptibility to certain cancer types. Further studies incorporating subjects with different ethnic backgrounds combined with re-sequencing of the marked region and functional evaluations are warranted.

## Supporting Information

S1 Fig Funnel plot for publication bias of the *HOTAIR* rs920778 polymorphism and cancer risk under dominant model (Funnel plot with pseudo 95% confidence limits was used). (TIF)

S2 Fig Funnel plot for publication bias of the *HOTAIR* rs4759314 polymorphism and cancer risk under dominant model (Funnel plot with pseudo 95% confidence limits was used). (TIF)

S3 Fig Funnel plot for publication bias of the *HOTAIR* rs1899663 polymorphism and cancer risk under dominant model (Funnel plot with pseudo 95% confidence limits was used). (TIF)

S1 Table List of excluded articles and their reasons for exclusion. (DOCX)

S2 Table Distributions of the genotypes and alleles of the *HOTAIR* rs920778 polymorphism. (DOCX)

S3 Table Distributions of the genotypes and alleles of the *HOTAIR* rs4759314 polymorphism. (DOCX)

S4 Table Distributions of the genotypes and alleles of the *HOTAIR* rs1899663 polymorphism. (DOCX)

S5 Table Newcastle-Ottawa quality assessment scale for each included study. (DOCX)

S6 Table Sensitivity analysis of rs920778 in dominant model. (DOCX)

S7 Table Functional annotation for the marker SNP rs920778 and the SNPs in strong linkage disequilibrium with the marker SNP. (DOCX)
