# Supplementary material for: Quantitative Assessment of the Polymorphisms in the HOTAIR lncRNA and Cancer Risk: A Meta-Analysis of 8 Case-Control Studies
Source: PLoS One. 2016 Mar 24;11(3):e0152296. doi: 10.1371/journal.pone.0152296 (PMC4806879; doi:10.1371/journal.pone.0152296)
Supplement: S1 Table — (DOCX) [file pone.0152296.s004.docx]

**S1 Table. List of excluded articles and their reasons for exclusion**

| Number | First Author | Year | Country | Reasons of excluding |
| --- | --- | --- | --- | --- |
| 1 | Bayram | 2015 | Turkey | not involving the polymorphisms of rs920778, rs4759314 or rs1899663 |
| 2 | Song | 2015 | China | not involving polymorphisms of *HOTAIR* |
| 3 | Tao | 2015 | China | not involving polymorphisms of *HOTAIR* |
| 4 | Chen | 2015 | China | not involving polymorphisms of *HOTAIR* |
| 5 | Zhuang | 2015 | China | not involving polymorphisms of *HOTAIR* |
| 6 | Chiyomaru | 2014 | USA | not involving polymorphisms of *HOTAIR* |
