# Supplementary material for: Quantitative Assessment of the Polymorphisms in the HOTAIR lncRNA and Cancer Risk: A Meta-Analysis of 8 Case-Control Studies
Source: PLoS One. 2016 Mar 24;11(3):e0152296. doi: 10.1371/journal.pone.0152296 (PMC4806879; doi:10.1371/journal.pone.0152296)
Supplement: S2 Table — (DOCX) [file pone.0152296.s005.docx]

**S2 Table. Distributions of the genotypes and alleles of the *HOTAIR* rs920778 polymorphism**

| Number | First Author | Case/Control | Frequency distributions of the genotypes | | | | | |
| --- | --- | --- | --- | --- | --- | --- | --- | --- |
|  |  |  | Case | | | Control | | |
|  |  |  | CC | CT | TT | CC | CT | TT |
|  |  |  | N(%) | N(%) | N(%) | N(%) | N(%) | N(%) |
| 1 | Bayram | 104/209 | 20(1.27) | 52(3.71) | 32(4.92) | 38(1.60) | 105(6.23) | 66(12.55) |
| 2 | Pan | 800/1600 | 420(26.68) | 321(22.90) | 59(9.06) | 980(41.28) | 575(34.12) | 45(8.56) |
| 3 | Yan | 502/504 | 12(0.76) | 151(10.77) | 339(52.07) | 18(0.76) | 190(11.28) | 296(56.27) |
| 4 | Bayram | 123/122 | 31(1.97) | 52(3.71) | 40(6.14) | 15(0.63) | 66(3.92) | 41(7.79) |
| 5 | Zhang | 2098/2150 | 1091(69.31) | 826(58.92) | 181(27.80) | 1323(55.73) | 749(44.45) | 78(14.83) |
