# Supplementary material for: Quantitative Assessment of the Polymorphisms in the HOTAIR lncRNA and Cancer Risk: A Meta-Analysis of 8 Case-Control Studies
Source: PLoS One. 2016 Mar 24;11(3):e0152296. doi: 10.1371/journal.pone.0152296 (PMC4806879; doi:10.1371/journal.pone.0152296)
Supplement: S3 Table — (DOCX) [file pone.0152296.s006.docx]

**S3 Table. Distributions of the genotypes and alleles of the *HOTAIR* rs4759314 polymorphism**

| Number | First Author | Case/Control | Frequency distributions of the genotypes | | | | | |
| --- | --- | --- | --- | --- | --- | --- | --- | --- |
|  |  |  | Case | | | Control | | |
|  |  |  | AA | AG | GG | AA | AG | GG |
|  |  |  | N(%) | N(%) | N(%) | N(%) | N(%) | N(%) |
| 1 | Zhang | 2098/2150 | 917(18.75) | 81(13.11) | 2(12.50) | 910(15.34) | 89(12.75) | 1(3.85) |
| 2 | Guo | 515/654 | 461(9.43) | 53(8.58) | 1(6.25) | 589(9.93) | 64(9.17) | 1(3.85) |
| 3 | Pan | 800/1600 | 451(9.22) | 48(7.77) | 1(6.25) | 914(15.41) | 83(11.89) | 3(11.54) |
| 4 | Du | 1275/1646 | 1083(22.14) | 186(30.10) | 6(37.50) | 1464(24.68) | 172(24.64) | 8(30.77) |
| 5 | Xue | 1734/1855 | 1528(31.24) | 200(32.36) | 5(31.25) | 1608(27.10) | 236(33.81) | 11(42.31) |
| 6 | Yan | 502/504 | 451(9.22) | 50(8.09) | 1(6.25) | 448(7.55) | 54(7.74) | 2(7.69) |
