# Supplementary material for: Quantitative Assessment of the Polymorphisms in the HOTAIR lncRNA and Cancer Risk: A Meta-Analysis of 8 Case-Control Studies
Source: PLoS One. 2016 Mar 24;11(3):e0152296. doi: 10.1371/journal.pone.0152296 (PMC4806879; doi:10.1371/journal.pone.0152296)
Supplement: S4 Table — (DOCX) [file pone.0152296.s007.docx]

**S4 Table. Distributions of the genotypes and alleles of the *HOTAIR* rs1899663 polymorphism**

| Number | First Author | Case/Control | Frequency distributions of the genotypes | | | | | |
| --- | --- | --- | --- | --- | --- | --- | --- | --- |
|  |  |  | Case | | | Control | | |
|  |  |  | GG | GT | TT | GG | GT | TT |
|  |  |  | N(%) | N(%) | N(%) | N(%) | N(%) | N(%) |
| 1 | Zhang | 2098/2150 | 725(50.35) | 256(48.95) | 19(48.72) | 724(43.67) | 250(32.22) | 26(37.14) |
| 2 | Yan | 502/504 | 339(23.54) | 149(28.49) | 14(35.90) | 326(19.66) | 158(20.36) | 20(28.57) |
| 3 | Pan | 800/1600 | 376(26.11) | 118(22.56) | 6(15.38) | 608(36.67) | 368(47.42) | 24(34.29) |
