# Supplementary material for: Quantitative Assessment of the Polymorphisms in the HOTAIR lncRNA and Cancer Risk: A Meta-Analysis of 8 Case-Control Studies
Source: PLoS One. 2016 Mar 24;11(3):e0152296. doi: 10.1371/journal.pone.0152296 (PMC4806879; doi:10.1371/journal.pone.0152296)
Supplement: S5 Table — (DOCX) [file pone.0152296.s008.docx]

**S5 Table. Newcastle-Ottawa quality assessment scale for each included study.**

| Studies | Selection | | | |  | Comparability | |  | Exposure | | | Total quality score |
| --- | --- | --- | --- | --- | --- | --- | --- | --- | --- | --- | --- | --- |
|  | Case definition adequate | Representativeness of the cases | Selection of controls | Definition of controls |  | Adjustment for age | Adjustment for lifestyle/traditional risk factors |  | Ascertainment of exposure | Uniform method of ascertainment | Non-response rate |  |
| Zhang 2014^[17]^ | 1 | 1 | 1 | 1 |  | 1 | 1 |  | 0 | 1 | 0 | 7 |
| Bayram 2015^[19]^ | 1 | 1 | 0 | 1 |  | 1 | 1 |  | 0 | 1 | 0 | 6 |
| Pan 2015^[16]^ | 1 | 1 | 1 | 1 |  | 1 | 1 |  | 0 | 1 | 0 | 7 |
| Xue 2015^[20]^ | 1 | 1 | 1 | 1 |  | 1 | 1 |  | 0 | 1 | 0 | 7 |
| Du 2015^[18]^ | 1 | 1 | 0 | 1 |  | 1 | 1 |  | 0 | 1 | 0 | 6 |
| Guo 2015^[15]^ | 1 | 1 | 1 | 1 |  | 1 | 1 |  | 0 | 1 | 0 | 7 |
| Bayram 2015^[14]^ | 1 | 1 | 0 | 1 |  | 1 | 1 |  | 0 | 1 | 0 | 6 |
| Yan 2015^[21]^ | 1 | 1 | 1 | 1 |  | 1 | 1 |  | 0 | 1 | 0 | 7 |
