# Supplementary material for: Quantitative Assessment of the Polymorphisms in the HOTAIR lncRNA and Cancer Risk: A Meta-Analysis of 8 Case-Control Studies
Source: PLoS One. 2016 Mar 24;11(3):e0152296. doi: 10.1371/journal.pone.0152296 (PMC4806879; doi:10.1371/journal.pone.0152296)
Supplement: S6 Table — (DOCX) [file pone.0152296.s009.docx]

**S6 Table. Sensitivity analysis of rs920778 in dominant model**

| Study omitted | Cancer type | OR (95%CI) | *P* | *P* for heterogeneity | *I^2^* |
| --- | --- | --- | --- | --- | --- |
| Zhang (2014) | ESCC | 0.99 (0.57-1.71) | 0.977 | 0.004 | 77.6% |
| Pan (2015) | gastric cancer | 1.00 (0.57-1.75) | 0.995 | 0.002 | 79.8% |
| Yan (2015) | breast cancer | 1.16 (0.86-1.56) | 0.344 | 0.002 | 79.9% |
| Bayram (2015) | gastric cancer | 1.25 (0.93-1.66) | 0.136 | 0.004 | 77.1% |
| Bayram (2015) | breast cancer | 1.44 (1.31-1.59) | <0.001 | 0.536 | 0.0% |
