# Supplementary material for: Quantitative Assessment of the Polymorphisms in the HOTAIR lncRNA and Cancer Risk: A Meta-Analysis of 8 Case-Control Studies
Source: PLoS One. 2016 Mar 24;11(3):e0152296. doi: 10.1371/journal.pone.0152296 (PMC4806879; doi:10.1371/journal.pone.0152296)
Supplement: S7 Table — (DOCX) [file pone.0152296.s010.docx]

| **S7 Table. Functional annotation for the marker SNP rs920778 and the SNPs in strong linkage disequilibrium with the marker SNP** | | | | | | | |
| --- | --- | --- | --- | --- | --- | --- | --- |
| SNP | marker SNP | *r*^2^ | eQLT | Open chromatin | miRNA binding | protein binding |  |
| rs920778 | rs920778 | 1 |  | Open chromatin |  | EZH2、CTBP2、CHD1、ZNF143、SUZ12 |  |
| rs10783617 | rs920778 | 1 |  |  |  | EZH2 |  |
| rs10783616 | rs920778 | 1 |  |  |  | EZH2 |  |
| rs4759313 | rs920778 | 1 |  |  |  |  |  |
| rs7958904 | rs920778 | 1 |  | Open chromatin | hsa-miR-615-3p | EZH2、TCF7L2 |  |
| rs2366151 | rs920778 | 1 |  |  |  | EZH2 |  |
| rs2002472 | rs920778 | 1 |  | Open chromatin |  | EZH2、SUZ12、CTCF、RAD21 |  |
| rs10783618 | rs920778 | 1 | *HOTAIR* |  |  | EZH2、SUZ12 |  |
| rs11170775 | rs920778 | 1 | *HOTAIR* |  |  | SUZ12 |  |
| rs4759059 | rs920778 | 1 | *HOTAIR* | Open chromatin |  | EZH2、SUZ12、YY1 |  |
| rs4237809 | rs920778 | 1 | *HOTAIR* | Open chromatin |  | SUZ12、YY1 |  |
| rs2366150 | rs920778 | 1 | *HOTAIR* |  |  |  |  |
